# Recurrent arterial ischemic strokes in a patient with patent foramen ovale and ductus arteriosus: Presentation of our management and review of the literature

**DOI:** 10.21542/gcsp.2019.13

**Published:** 2019-09-20

**Authors:** Dimitrios Panagopoulos, Sofia Loukopoulou, Evangelos Karanasios, Nikolaos Eleftherakis

**Affiliations:** 1 Neurosurgical Department of Pediatric Hospital of Athens, ‘Agia Sophia’, Thivon & Papadiamantopoulou St, Goudi, 11527 Athens, Greece; 2 Cardiology Department of Pediatric Hospital of Athens, ‘Agia Sophia’, Thivon & Papadiamantopoulou St, Goudi, 11527 Athens, Greece

## Abstract

Ischemic stroke in children is a relatively rare entity, relative to the adult population. The most common potential risk factors include cardiac embolism, prothrombotic states and vasculopathies. The diagnosis is concerning for the need to identify the underlying cause. Treatment of the proximate source of ischemia can often protect against future events.

We present the case of a 7-year-old patient who initially presented with an ischemic brain insult which was repeated, despite the initiation of anticoagulation therapy. The investigation revealed patent foramen ovale and patent ductus arteriosus and because of the recurrent ischemic ictuses, transcatheter closure of both defects was decided. A brief description of the literature is also presented.

## Introduction

Pediatric arterial ischemic stroke (AIS) is an important cause of neurologic morbidity in children. While guidelines have been published regarding the evaluation and management of stroke in children, these are largely consensus-based and not usually supported by strong evidence.

Estimates of the incidence of childhood AIS are variable and highly dependent on the search strategy employed as well as the study population. The largest of such studies found an incidence of pediatric AIS of 1.2 per 100,000 person-years. Cryptogenic (of unknown cause) ischemic strokes are now estimated to represent about 25% of all ischemic strokes. Most cryptogenic strokes are nowadays thought to represent thromboembolic insults (ESUS)^1^. Children with cardiac disease represent one of the most significant subsets of pediatric AIS patients. Across most series, cardiac risk factors are present in 2–31% of children with AIS.

A patent foramen ovale (PFO) is a normal connection between the right and left atria, caused by the incompetence of the fossa ovalis valve during fetal life^2^. The shunt is usually right to- left despite the gradient pressure between the atria. The connection closes in the majority of people over a period of time after birth. However, if the septum primum fails to fuse with the septum secundum, the PFO remains patent, allowing interatrial blood flow in approximately 25% of the adult population^3,4^.

The role of a PFO or other potential intracardiac shunts in stroke or stroke recurrence in childhood is unclear^5^. PFO has been implicated in multiple disease states^6^. Some have described it as a causative agent, whereas others describe it as an innocent bystander^7^.

The cause remains undetermined after evaluation has excluded large cerebral artery occlusive disease, small vessel disease (lacunes), and cardiac emboli^8,9^. The thrombus is thought to originate from one of the well-established potential embolic sources, including cardiac origin (e.g., mitral annular calcification), deep venous system via paradoxical embolism, and the cerebral vasculature, arising from the cervical of carotid arteries^10^. The usual evaluation includes MRI of the brain, MRA of the neck, echocardiography and ECG monitoring^11^.

The statistical association between cryptogenic stroke and the presence of a PFO has long been established^11^. Although a PFO can be demonstrated in approximately 20% to 25% of the general population, it is much more frequent in patients who have suffered a cryptogenic stroke. In a series of contemporary experiences, the prevalence of PFO in cryptogenic stroke ranged from 21% to 63%^11,12^. Other studies showed that PFO can be the offending mechanism in up to 40% of patients suffering ESUS^13^. Paradoxic embolism from the venous system, or from thrombus formed in situ, has been presumed to be the causal mechanisms for this association.

**Figure 1A.**
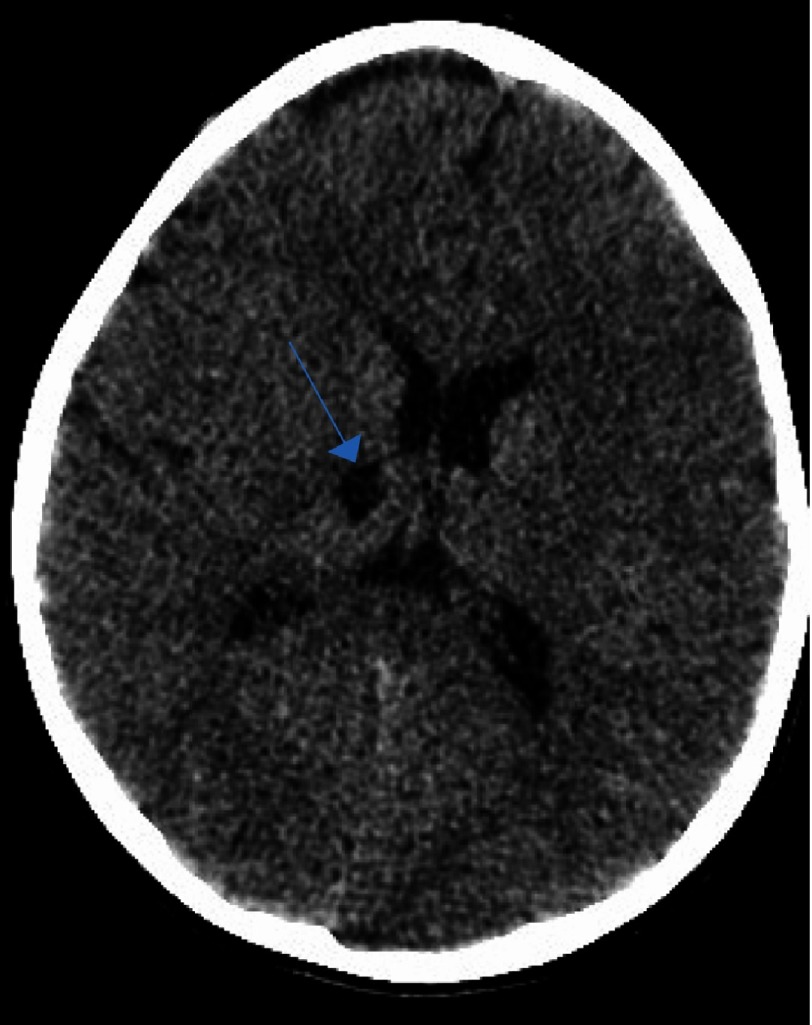
CT scan after the first ischemic insult. Note the hypointensity signal area at the limit between the genu and the posterior limp of the internal capsule of the right basal ganglia (arrow).

**Figure 1B.**
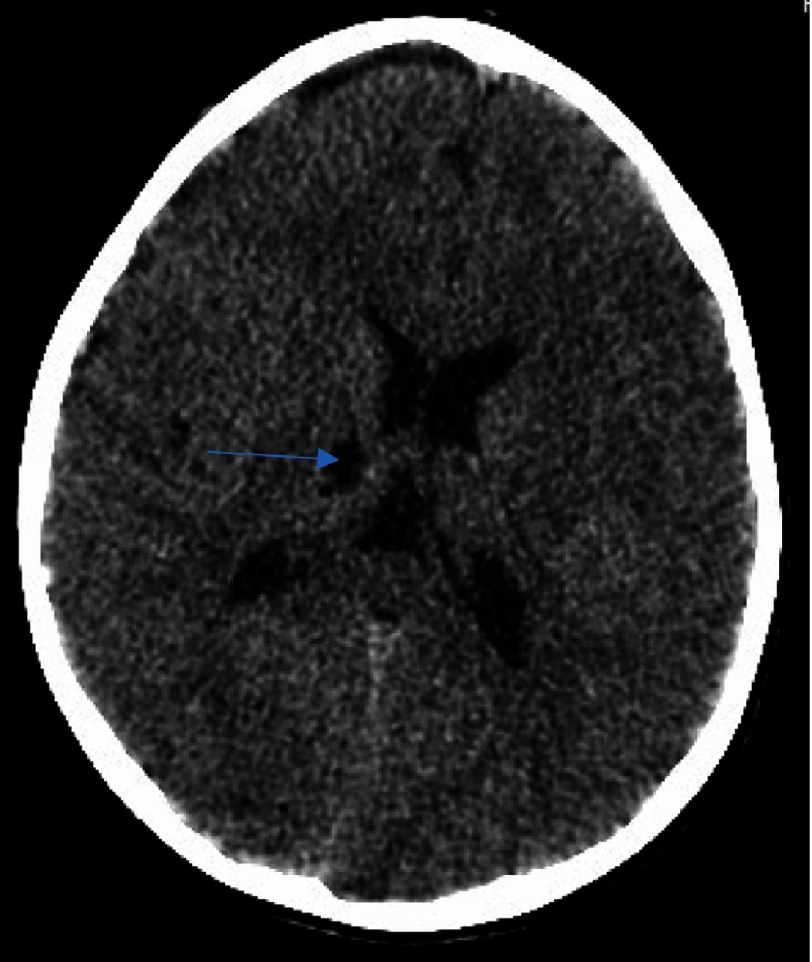
As in Figure 1A, with extension toward the ipsilateral thalamus (arrow).

**Figure 1C.**
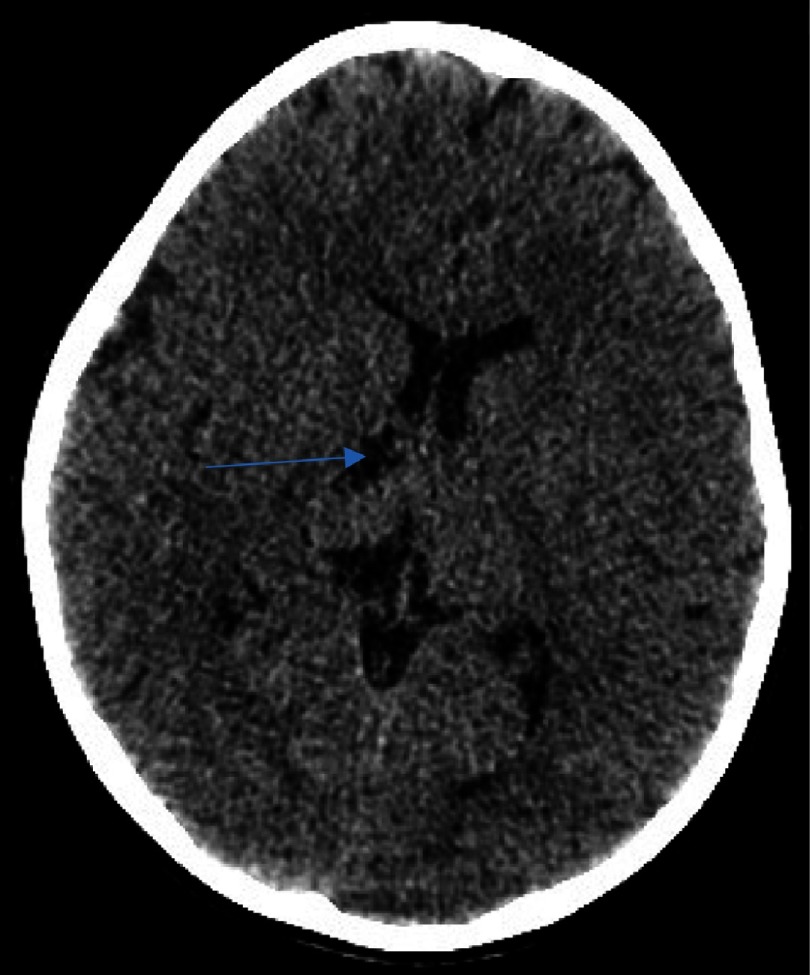
Delineating extension in the infarct in the globus pallidus territory (arrow).

## Case description

We describe the case of a 7-year-old male who presented with a first episode of arterial ischemic stroke. The neurological evaluation revealed a left sided hemiparesis which was not involving the facial musculature. The initial CT scan revealed an area of hypointensity in the region of the right basal ganglia. More specifically, its anatomical contribution was extending to the anterior limb and the genu of the right internal capsule, and in the nearby territory of the globus pallidus. This infarct refers to the anatomical contribution of the medial and lateral lenticulostriate arteries and was attributed possibly due to the migration of an arterial thrombus to their vessel of origin (Figures 1A–1C).

The diagnostic work up for the recognition of the underlying cause of the AIS included the investigation of possible prothrombotic conditions and thrombophilia, but it did not reveal any underlying pathologic condition. The patient underwent a detailed cardiologic work up, which included a transesophageal echocardiographic examination. It revealed the presence of a patent foramen ovale (capillary subtype) and a patent ductus arteriosus (Figures 2A, 2B).

**Figure 2A.**
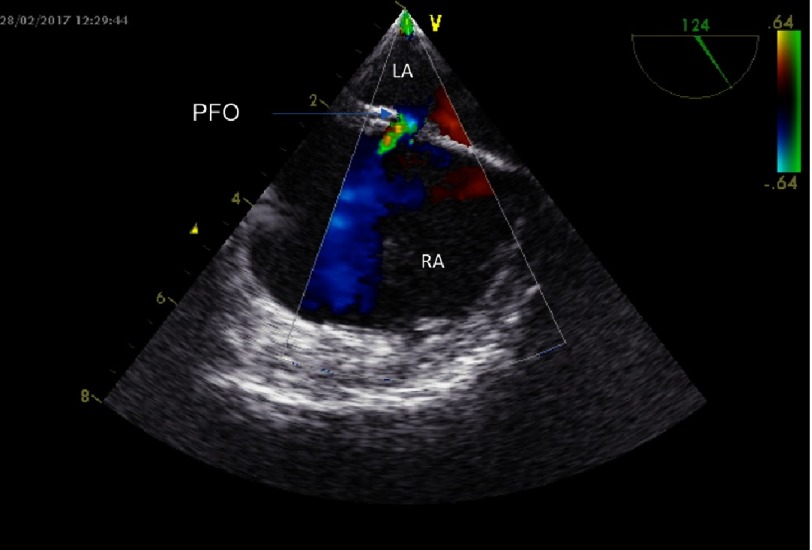
Echocardiographic illustration by transesophageal echocardiography of the small patent foramen ovale (arrow) before closure. LA, Left Atrium; RA, Right Atrium; PFO, Patent Foramen Ovale.

**Figure 2B.**
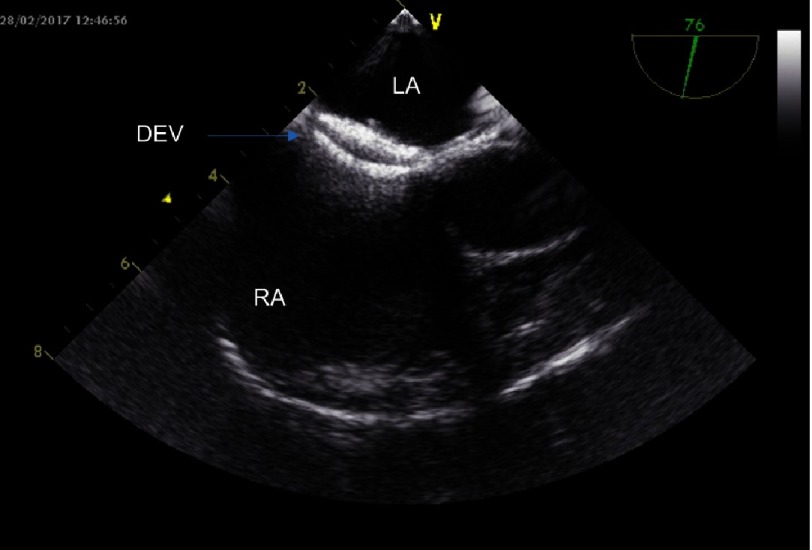
Patent foramen ovale closure after deployment and detachment of the Amplatzer PFO Cribriform Ocluder illustated by transesophageal echocardiography. LA, Left Atrium; RA, Right Atrium; DEV, Amplatzer PFO Occluder device.

Based on these findings, the patient was immediately treated with anticoagulation therapy. Despite that, he developed a second episode of arterial ischemic stroke 9 months later. The neurological evaluation after the second ictus revealed complete left-sided hemiparesis with involvement of the musculature of the ipsilateral half of the face.

**Figure 3A.**
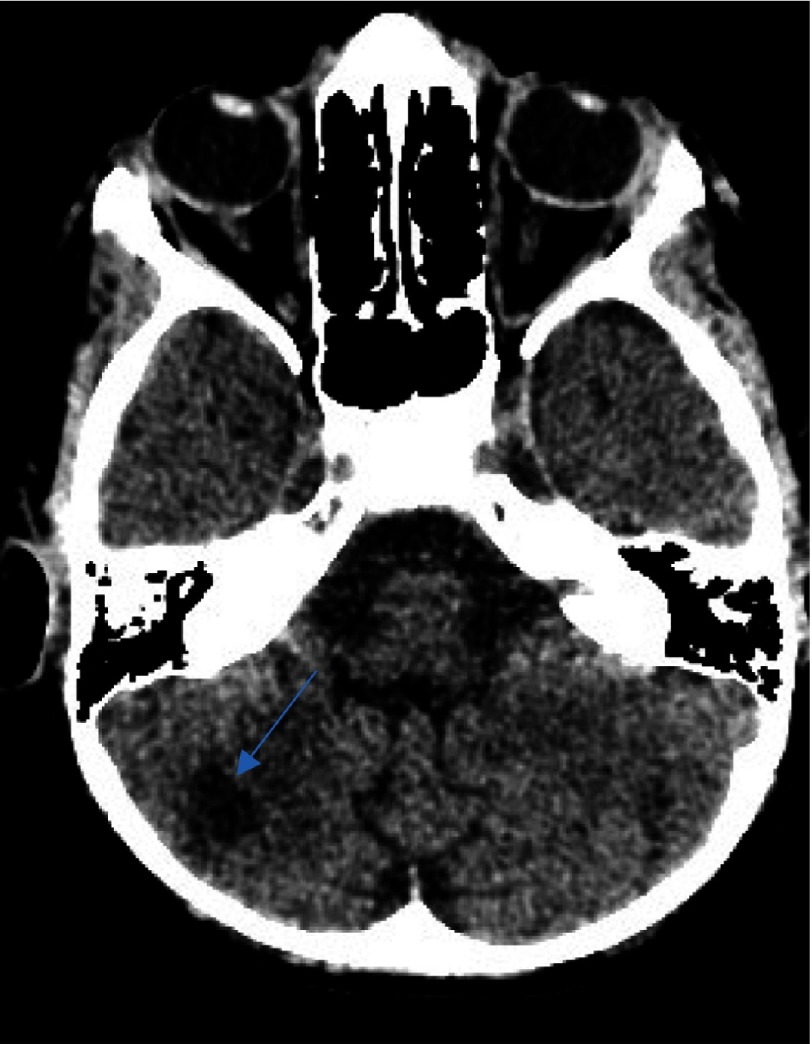
CT scan after the second ischemic insult. Note the hypointensity signal area at the depth of the right cerebellar hemisphere (arrow).

**Figure 3B.**
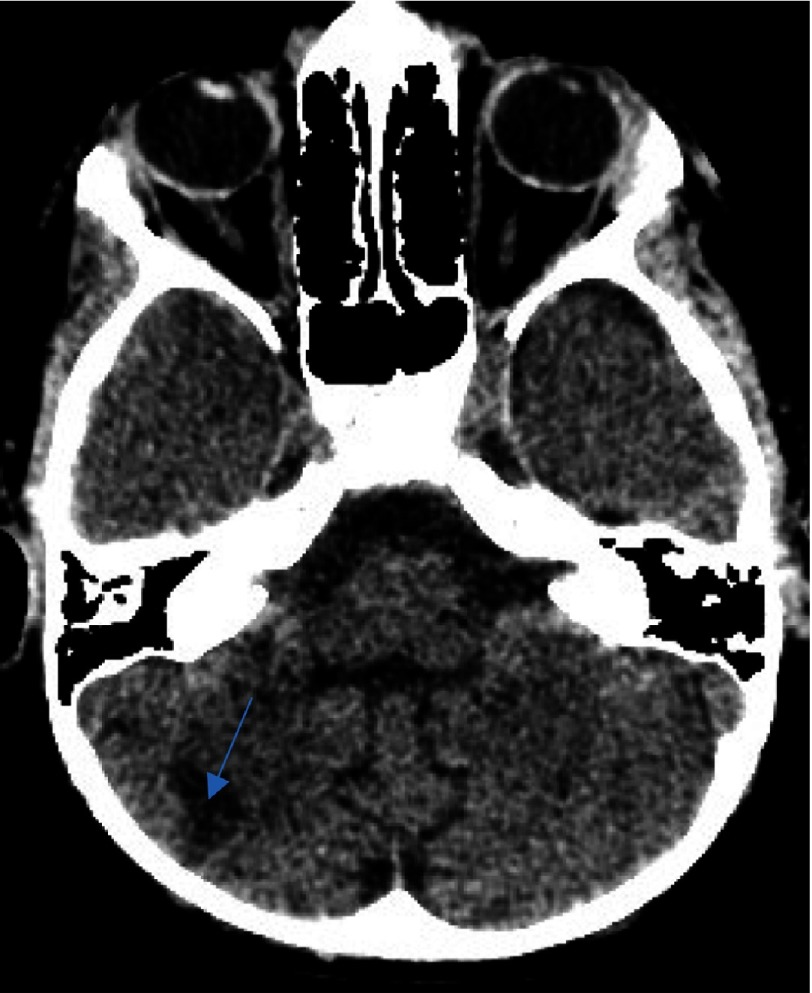
Same findings as Figure 3A, delineates the extent of the infarct (arrow). This infarct, due to its anatomical distribution, may be not clinically relevant.

**Figure 3C:**
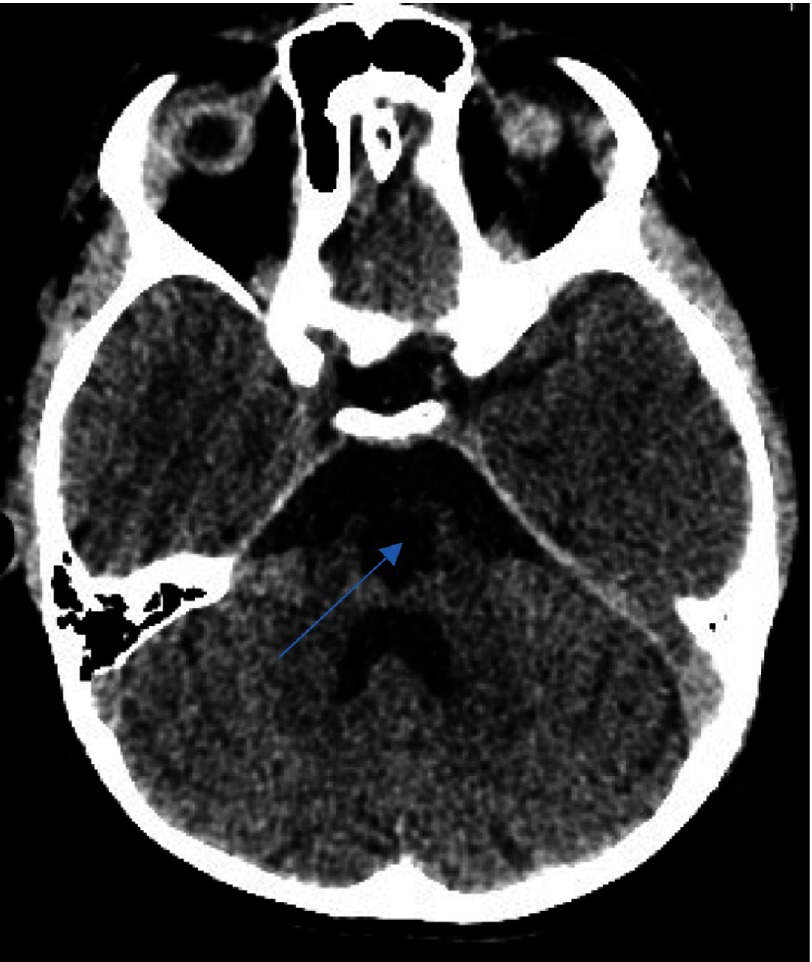
CT scan illustrating extensive ischemic infarction in the region of the pons (arrow).

**Figure 3D.**
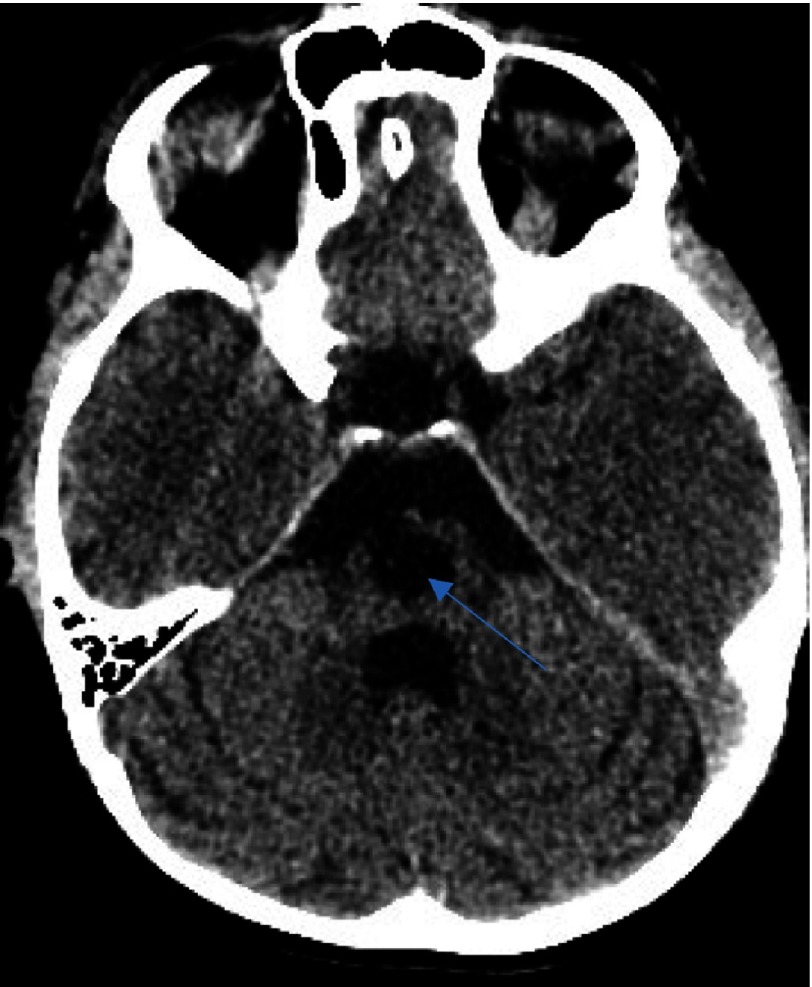
Same CT scan, at a higher level, visualizing infarction at the ponto-mesencephalic junction (arrow).

**Figure 3E.**
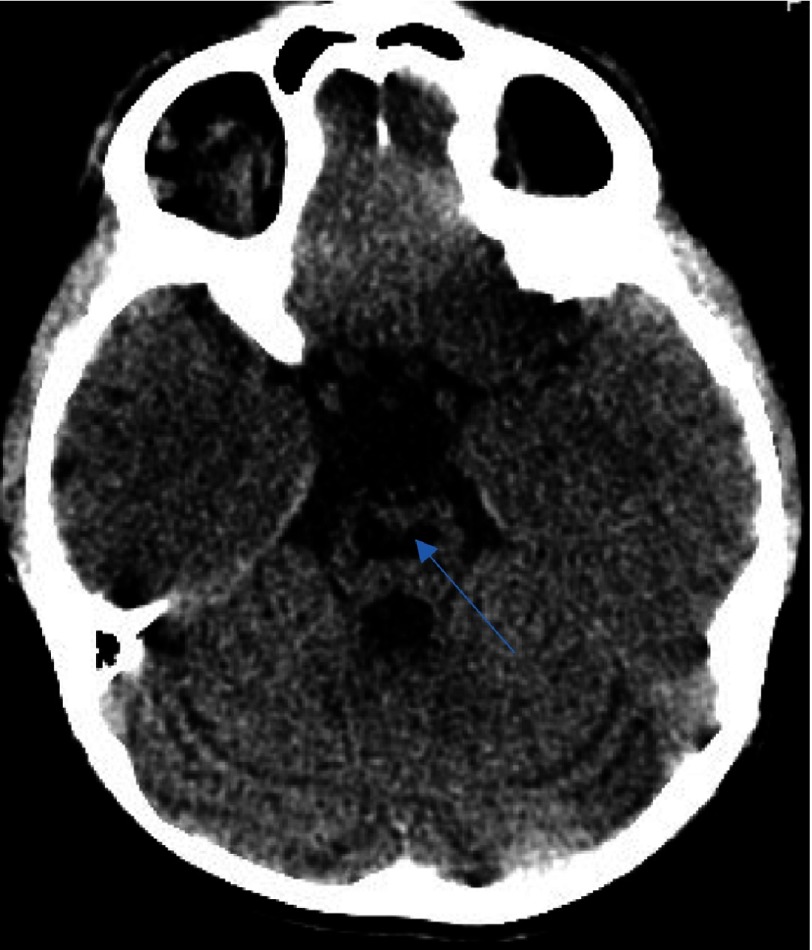
Axial CT scan at even higher level, verifying extension of the infarction into the mesencephalon (arrow).

He underwent a radiological evaluation with a new CT scan, which revealed an extensive area of hypointensity, extending to the mesencephalon-pontine region the dimensions of the basal ganglia infarction were significantly reduced. An additional new infarction territory was recognized, in the area of the right cerebellar hemisphere, lateral to the vermis. The anatomic substrate of the new stroke could be attributed to a second stroke that developed in the region that is nourished from the perforators of the basal artery, near its terminal bifurcation. The infarct with a hemispheric distribution was attributed to a thrombus-related obstruction of hemispheric branches of the vertebrobasilar arterial system (Figures 3A–3E).

After the second episode of AIS, we decided to occlude the foramen ovale with the use of the Amplatzer PFO Occluder Cribiform No 18, and by the way, of the patent ductus arteriosus with an MReye Flipper PDA Closure Detachable Coil IMWCE-8-PDA-4 Coil 8 mm.

We performed retrograde catheterization of the ascending aorta through the right femoral artery, utilizing the percutaneous technique. Through the patent ductus arteriosus, the catheter was inserted to the pulmonary artery. Insertion and detachment of the detachable coil from the arterial route followed (Figures 4A, 4B). Next step was the catheterization of the right cardiac cavities and of the pulmonary artery via the right femoral vein, using the percutaneous technique. The catheter is inserted into the left atrium and the left superior pulmonary vein, via the PFO. The system responsible for deployment of the Amplatzer PFO Ocluder was inserted via the transvenous route, and the ocluder was detached (Figures 5A, 5B), (Table 1). After the procedure, a gradual discontinuation of anticoagulant therapy was decided, which was followed by replacement with antiplatelet therapy, namely aspirin.

**Figure 4A.**
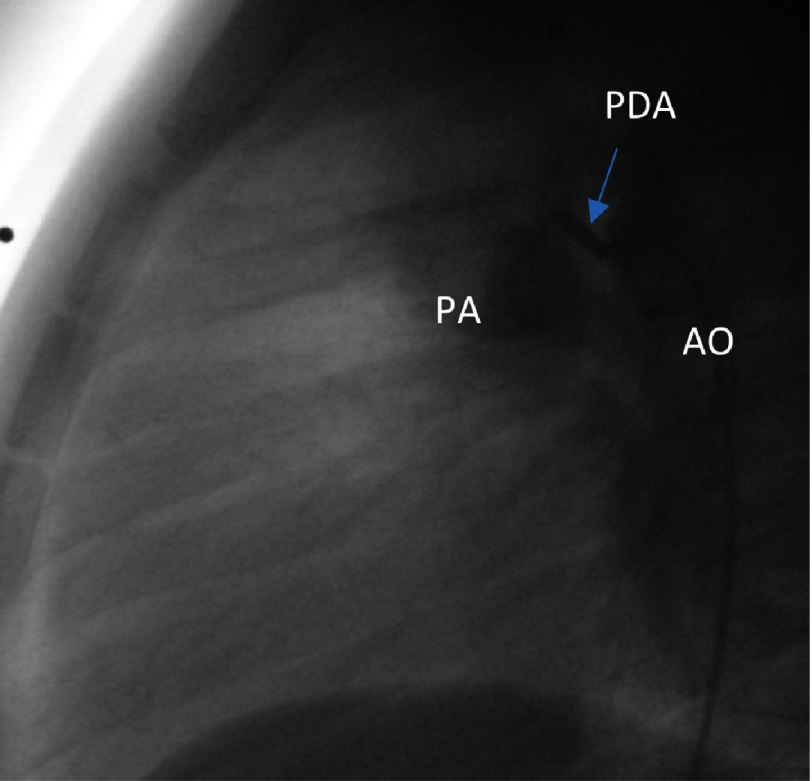
Angiographic illustration in lateral view of the small patent duct before closure with a detachable coil. AO, Aorta; PA, Pulmonary Artery; PDA, Patent Duct; COIL, Detachable Coil.

**Figure 4B.**
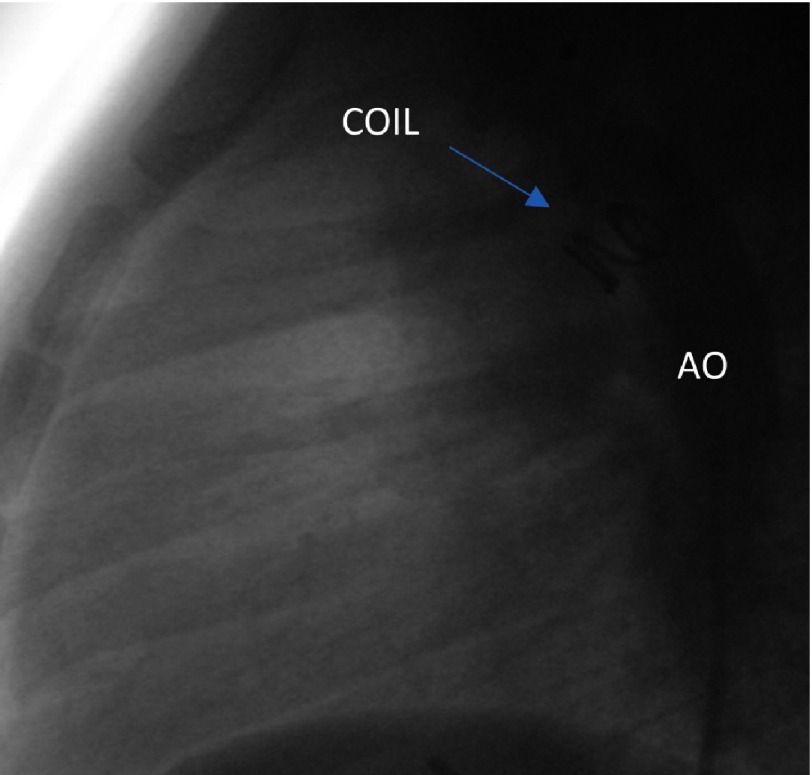
Angiographic illustration in lateral view of the small patent duct after closure with an MReye Flipper PDA Closure Detachable Coil IMWCE-8-PDA-4. AO, Aorta; PA, Pulmonary Artery; PDA, Patent Duct; COIL, Detachable Coil.

**Figure 5A.**
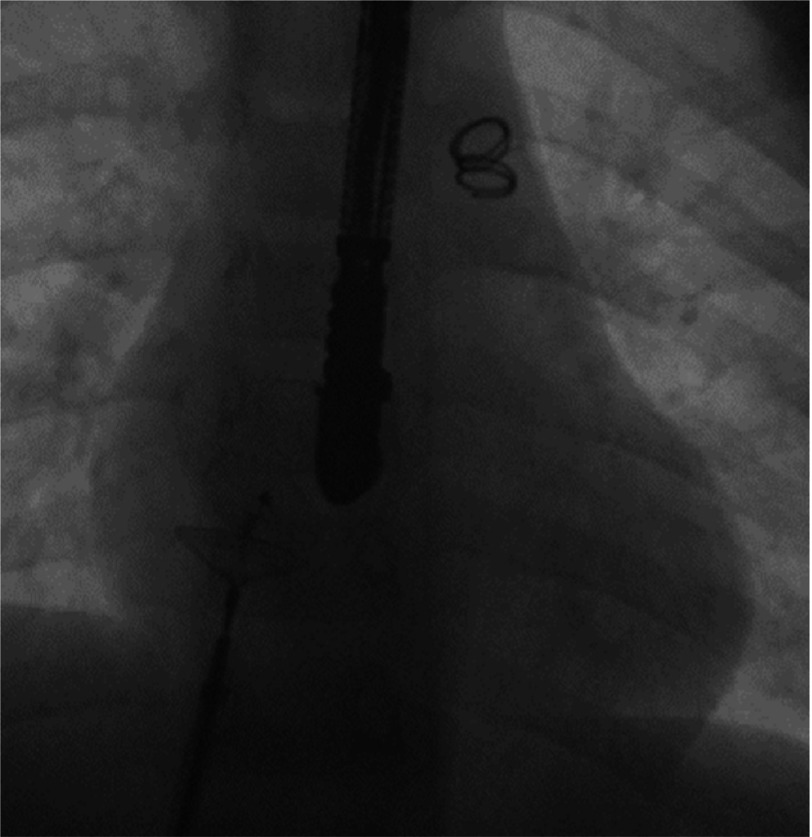
Patent foramen ovale closure after deployment but before detachment of the Amplatzer PFO Cribiform Ocluder No 18 in anteroposterior view under transesophageal echocardiographic guidance.

**Figure 5B.**
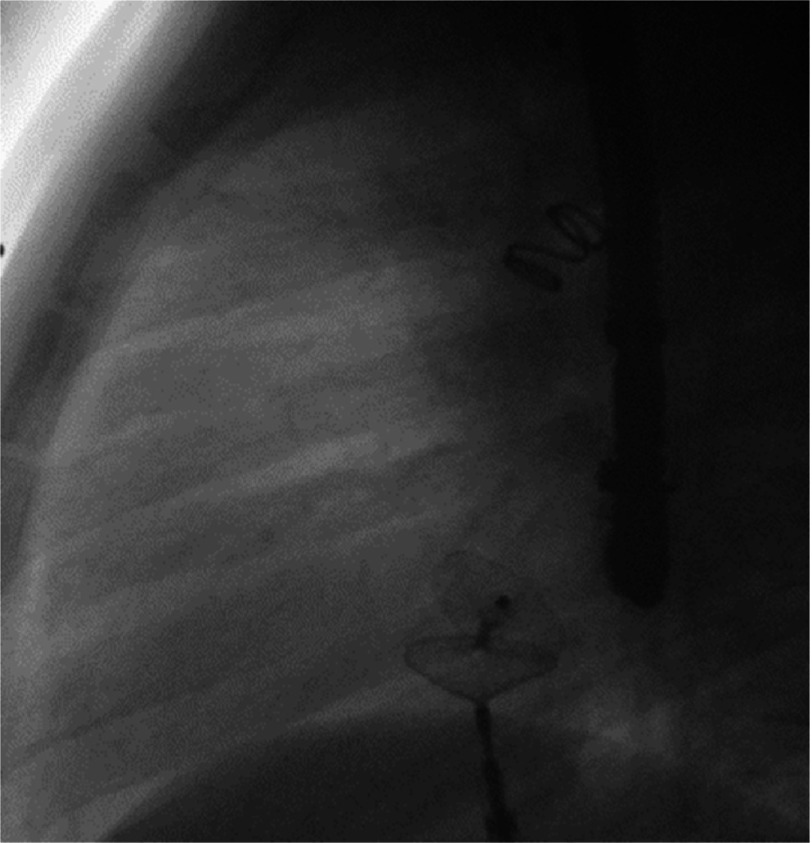
Patent foramen ovale closure after deployment but before detachment of the Amplatzer PFO Cribiform Ocluder in lateral view under transesophageal echocardiographic guidance.

The follow-up period extends to approximately one and a half years, during which neither adverse effects from the therapy were noted, nor any new ischemic strokes. The patient’s neurological status remains stable.

**Table 1:** Cardiac catheterization.

Catheter position	Pressures (mm Hg)			Saturation O_2_ (%)
	Systolic	Diastolic	Mean	
Inferior Vena Cava				82
Right Ventricle	25	3		
Pulmonary Artery	20	9	14	83
Pulmonary Vein				100
Ascending Aorta				99

## Discussion

With a high prevalence in the general population of approximately 25%, and a prevalence in the cryptogenic stroke population approaching 40%, the propensity of a PFO to precipitate or enable stroke, especially in young, otherwise healthy individuals, has been the subject of much debate^6^.

Nowadays, it has not been certified whether PFO is indeed a risk factor for stroke. Results about the association of PFO with first stroke^13,14^ and with recurrent stroke^15^ have been controversial.

The probability that a PFO incidentally identified during patient’s evaluation of an ESUS is etiologically related to the ictus or not, depends on the patient’s age, presence of traditional risk factors, and location of cerebral infarct^16^. Therefore, there have been efforts to isolate the specific patient characteristics that could be important in patient selection in therapeutic decision-making.

Other anatomic considerations that can contribute to PFO shunting characteristics include the tunnel length, the extent of malposition between the septa which is affected by atrial stretch of the septal limbus, and the presence and severity of an ASA^6,17^.

Another important issue is the possible association of stroke recurrence in patients with PFO-related stroke. Even though this risk is thought to be low^1^, the biological relevance of PFO is unknown. PFO could be the cause of silent brain infarcts in our case, two infarcts with clinical manifestations have occurred.

In a recent study^18^, the specific clinical and radiological characteristics for PFO patients presenting with cryptogenic embolic strokes were evaluated. PFO primarily consisted of younger patients with a relatively healthy risk factor profile and posterior distribution of stroke, which is in accordance with our patient characteristics, who had one stroke located in the posterior cerebral circulation territory.

The 2014 Guidelines for the Prevention of Stroke in Patients with Stroke and Transient Ischemic Attack^7^ recommend antiplatelet therapy in patients with an ischemic stroke or TIA and a PFO who are not undergoing anticoagulation therapy (Class I; Level of Evidence B); for patients with an ischemic stroke or TIA and both a PFO and a venous source of embolism, anticoagulation is indicated, depending on stroke characteristics (Class I; Level of Evidence A)^7,19^.

Another study^8^ demonstrated that the appropriate treatment for patients with cryptogenic stroke and a patent foramen ovale depends upon the presence or absence of venous thromboembolism. If venous thromboembolism is present the appropriate treatment is the same as for pulmonary embolism; anticoagulation^19^. If there is no evidence of venous thromboembolism the appropriate treatment is antiplatelet therapy.

A non-randomized comparison of 308 cryptogenic stroke patients and PFO who underwent percutaneous closure with those that received medical treatment alone suggested that PFO closure may be especially beneficial in patients who have had more than one event in the past, and may represent the highest-risk group^20^.

The American College of Chest Physicians evidence-based clinical practice guidelines recommend surgical closure of a PFO RTLS if an AIS occurs secondary to cardio-embolic causes but the recommendation stems from low or very low-quality evidence^2,21^.

A multidisciplinary Italian task force on the management of patients with a PFO and cryptogenic stroke recommends that those with an initial or recurrent ischemic event while on medical therapy (antiplatelet or anticoagulants) should be offered transcatheter closure of the PFO^22^.

Closure of the PFO may also be considered in patients with an initial stroke who have one or more anatomical (atrial septal aneurysm, large PFO >4 mm, Eustachian valve >10 mm, long PFO tunnel) or clinical (recurrent stroke, multiple ischemic lesions radiologically, thrombophilia, deep venous thrombosis) risk factors with the understanding that the procedure may not prevent recurrence within two years^22^.

Even if medical therapy is considered efficacious, the feasibility of lifelong medical therapy, monitoring of anticoagulation levels^23^ and compliance with medical regimens in young patients is poor.

A study compared PFO closure to medical treatment in a non-randomized observational study on 308 young adults with cryptogenic stroke^20^. The combined end points of death, stroke, or TIA were significantly lower after percutaneous PFO closure than with antiplatelet therapy. Percutaneous PFO closure had a higher efficacy in preventing recurrence in patients with multiple cerebrovascular events (7.3% compared to 33.2% in the medical treatment arm, p=0.01). This is in concordance with our case, as the patient suffered at least three cerebrovascular ischemic insults. The results of this study are limited by the nonrandomized design, which may have introduced bias.

PFO and atrial septal aneurysm in association with cryptogenic stroke may result in higher recurrence rates and multidisciplinary task force recommendations support PFO closure in such cases with the full understanding that there may be a recurrence within two years post-procedure. Surgical closure may also be offered to patients who have recurrent cryptogenic ischemic events while on well-controlled cryptogenic ischemic events while on well-controlled medical therapy, with the proviso that they have been thoroughly counselled on the risk of recurrence.

A 5-year extended follow-up of the RESPECT cohort was recently presented^24^. This long-term follow-up becomes especially important when event rates are low and when considering secondary prevention in relatively young patient populations. Based on a blinded assessment of recurrent cryptogenic stroke in the intention-to-treat group, there was a 54% relative risk reduction in favor of PFO closure compared with medical therapy alone.

To date, three large, multicenter randomized controlled trials have been published examining PFO closure in the secondary prevention of stroke entitled:

(1) Closure or Medical Therapy for Cryptogenic Stroke with Patent Foramen Ovale (CLOSURE),

(2) Percutaneous Closure of Patent Foramen Ovale in Cryptogenic Embolism (PC trial), and

(3) Closure of Patent Foramen Ovale versus Medical Therapy after Cryptogenic Stroke (RESPECT)^1,25^.

Numerous meta-analyses have been performed combining the data of the three published trials (25). The majority of these studies have suggested a conglomerate benefit for PFO closure compared with medical therapy with the composite outcome and with stroke/TIA.

The first pooled analysis of individual patient data from randomized controlled trials^26^ suggested that although PFO closure was not statistically significant in the composite outcome measure, the outcome of stroke was statistically significant in all comparisons. Case series and meta-analyses studies have compared the use of a percutaneously placed PFO occlusive device with best medical therapy alone for prevention of recurrent ischemic neurologic symptoms—the vast majority of them demonstrate low rates of adverse effects and recurrence of neurological ictuses after PFO closure^27^.

## Conclusions

Cryptogenic stroke is one of the several pathologic processes PFO is implicated. Indeed, it has a higher prevalence of PFO (40%–50% incidence) compared with the general, not affected population (20%–25% incidence).

Stroke in association with a PFO may be due to paradoxical embolization via a right to left intracardiac shunt^2^, but the exact contribution of PFO to stroke or stroke recurrence in childhood remains unclear. Paradoxical embolism from a PFO as a cause of transient ischemic attack or stroke is a diagnosis of exclusion.

Although transcatheter PFO closure for cryptogenic stroke has been in use for more than 2 decades, indications and patient selection have remained controversial (7). Collaborative, observational studies have provided insight into risk-stratification to identify patients in whom PFO closure may provide the greatest potential. According to studies, it seems to be reasonable that patients with cryptogenic stroke, particularly those ≤60 years old and those who additionally have an ASA (atrial septal aneurysm), may benefit most from device (mechanical) closure of PFO^28–30^.

The United States Food and Drug Administration (FDA), in October 2016, approved the use of the Amplatzer PFO Occluder for percutaneous transcatheter closure of a PFO in order to reduce the risk of a recurrent ischemic CVA in patients predominately between ages of 18 and 60 years. Prerequisite was that patients would have suffered a verified cCVA due to presumed paradoxical embolism, in order to exclude other known causes of ischemic CVA^28^.

Long-term follow-up from randomized clinical studies has provided clarity regarding the efficacy of PFO closure in secondary stroke prevention.

Overall, high-quality evidence and robust recommendations for the management of a PFO in conjunction with a stroke in childhood are still awaited, pending the conduct of rigorously designed randomized clinical trials in neonates, children and adolescents^6^. For the first time in decades there is randomized controlled trial evidence supporting the observational data and physiologic hypothesis that PFO closure can help prevent recurrent stroke in appropriately-selected patients.

PFO is well known to be implicated in several pathologic processes, one them being cCVA. Recognition of patients most likely to have had a neurological ictus etiologically related to paradoxical embolism through a PFO is of paramount importance, as most recent trials identify “high-risk” PFOs as more likely to benefit more from percutaneous closure, compared to the best medical therapy alone^28^.

The rationale we present the current case is that we treated a patient with three documented ischemic events, which were attributed after extensive laboratory and clinical investigation to a PFO. Our treatment modality seems to be in concordance with the most recently adopted treatment policy of such situations, that is closure of the patent foramen ovale. This is augmented by the fact that although the patient was on anticoagulation therapy, the ischemic insults regressed.

## ABBREVIATIONS

AIS: arterial ischemic stroke
ESUS: embolic stroke of undetermined source
PFO: patent foramen ovale
MRI: magnetic resonance imaging
MRA: magnetic resonance angiography
ECG: electrocardiography
CT: computed tomography
ASA: atrial septal aneurysm
TIA: transient ischemic stroke
cCVA: cryptogenic stroke
